# A promising front in the war on inequality

**DOI:** 10.1073/pnas.2005162117

**Published:** 2020-05-04

**Authors:** David B. Grusky

**Affiliations:** ^a^Department of Sociology, Stanford University, Stanford, CA 94305

Over the last half century, earnings and income inequality have increased within many countries, although the timing and extent of the increase have been variable. This development has engendered a large stream of social science research that has successfully identified some of the main culprits behind the takeoff in inequality. Given that this topic has been worked for so long and with such success, it might be thought that the chances of uncovering an unusually important fact about trends in inequality are rather low. Although that may well be the case, the PNAS paper “Rising between-workplace inequalities in high-income countries” by Tomaskovic-Devey et al. (1) has evidently beaten the odds, breaking ground by showing that the between-workplace share of earnings inequality is rapidly growing in most well-off countries. The inequality regime of contemporary well-off countries melds together 1) superstar workplaces that are chock full of relatively high-earnings workers and 2) low-end workplaces that are chock full of relatively low-earnings workers.

This result may be understood as the analog to the well-known finding that residential segregation by income is increasing in the United States. As Reardon et al. (2) show, the United States is increasingly segregated into separate residential neighborhoods, some for high earners and others for low earners. It seems that economic segregation of all types is the new normal: We sleep by night in segregated neighborhoods and then work by day in segregated workplaces.

This latest finding on rising earnings inequality builds upon a preexisting country-specific literature showing that between-workplace or between-firm inequality accounts for a rising share of inequality in the United States (3), West Germany (4), and Sweden (5). The key contribution of Tomaskovic-Devey et al. (1) is to show that this result is a very common one that holds in 12 of 14 well-off countries. Moreover, Tomaskovic-Devey et al. (1) also show that between-workplace inequality is more prominent in the private sector than in the public sector, although it is frequently growing in both sectors.

These results stand as fundamental social facts around which much new research will hopefully be oriented. There are two lines of future research that, in particular, are especially important to pursue, the first pertaining to the causes of this development and the second to its consequences. It is important to understand the causes because otherwise one would be hard pressed to choose the most promising strategy to reverse the takeoff in earnings inequality. It is likewise important to understand the consequences because, whatever the causes may be, the rise of between-workplace inequality shapes the everyday life of workers and may change how they see the world and engage with it.

## The Causes of Rising Between-Firm Inequality

In the more speculative side of their article, Tomaskovic-Devey et al. (1) argue that any effort to reduce market income inequality requires policies that “raise the bargaining power of lower-skilled workers” and “target the increasing market power of firms in concentrated markets” (ref. 1, p. 2). This conclusion rests on evidence that between-workplace inequality tends to increase within countries that have weakened their capacity for collective bargaining or reduced their employment protections. These “institutional protections” make it difficult for employers to outsource employees, to globalize production, and to engage in other wage-reducing practices that entail relocating low-skill workers into separate firms or establishments.

This evidence on the causes of rising between-workplace inequality is valuable because it tells us how to reduce market inequality. The suggestive prescription coming out of the research of Tomaskovic-Devey et al. (1) is that we need to ramp up collective bargaining and employment protections. Although much attention of late has been given to after-market redistribution (e.g., tax policy), Tomaskovic-Devey et al. (1) thus offer here a predistributive reform that is attractive because it affects the sizes of the paychecks that workers bring home and does not, therefore, have the delegitimating taint of an after-market “handout.” Because workers often assume, however wrongly, that their paychecks reflect their marginal product, the most palatable reforms are those that are embedded in our labor market institutions and thus unobtrusively affect the size of their paychecks. These types of reforms, because they are buried deep in the innards of our markets, are naturalized in ways that render the resulting reductions in inequality less likely to be challenged and reversed.

This recommendation to reinstall institutional protections is attractive regardless of the veracity of the underlying causal claim. Even if Tomaskovic-Devey et al. (1) are wrong and the withering away of institutional protections is not a main cause of the takeoff in between-workplace inequality, it is still a worthy recommendation because it can get the job done without, as noted above, the endless contestation that after-market reforms can engender. As Art Goldberger (6) emphasized, we must not assume that the only way to undo effects is to address their causes, a point that he famously makes by noting that eyeglasses are a much-appreciated corrective even though nearsightedness is mainly a genetic disorder. The same point holds here: We can reasonably turn to institutional protections for the purpose of reducing inequality even if the loss of those protections was not, across all countries, invariably the cause of the increase in between-workplace inequality.

It would nonetheless be a yet more attractive reform were the loss of such protections indeed the key cause. In the best of all possible worlds, a cause-targeting reform is to be preferred, because it is more likely to continue to respond appropriately as conditions develop. Although a particular pair of eyeglasses may initially correct the effects of a genetic disorder, the eyeglasses will typically grow ever less useful as the disorder develops over time. We are thus left with the task of endless trips to the optometrist to chase after these unfolding implications.

**Fig. 1.**
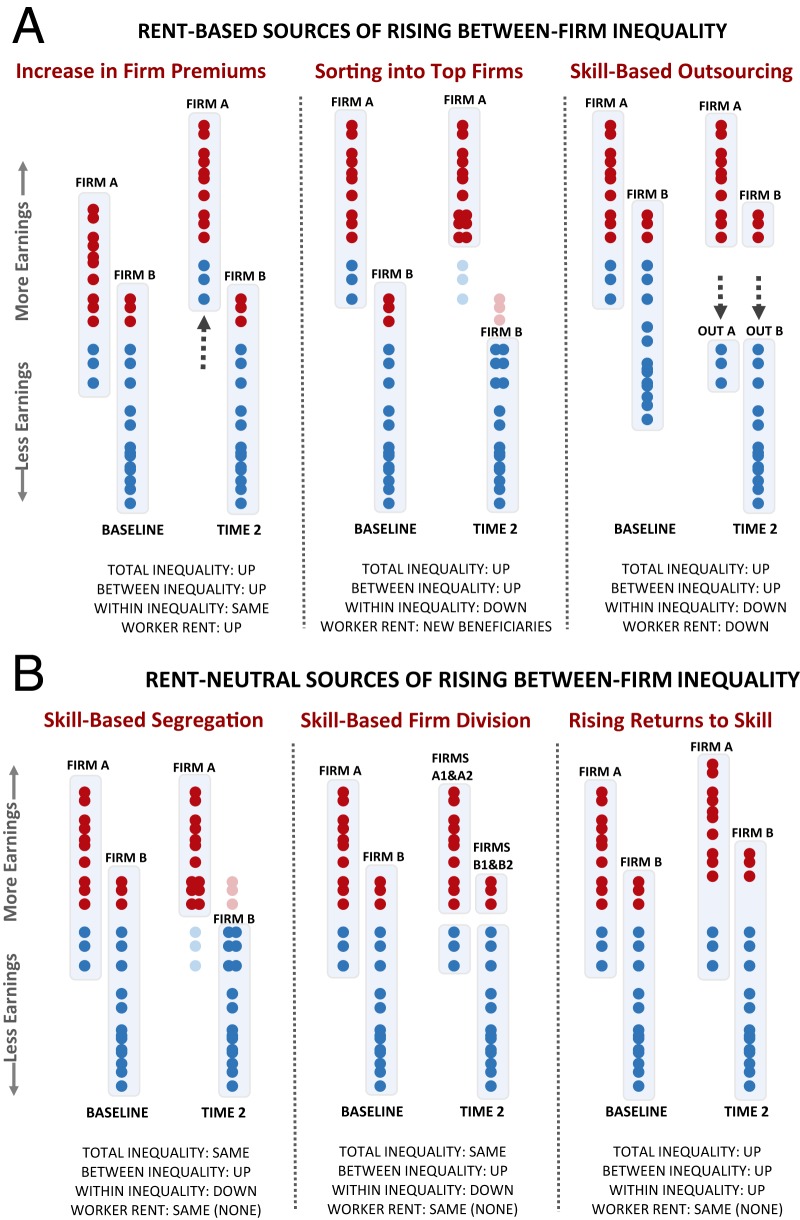
(*A*) Rent-based sources of rising between-firm inequality. (*B*) Rent-neutral sources of rising between-firm inequality.

This is all to say, then, that social scientists would do well to build on the important work of Tomaskovic-Devey et al. (1) by examining whether between-workplace inequality is indeed increasing because institutional protections have been lost (or were never in place). It is a difficult job not just because of the sheer number of possible causes but also because different causes are likely in play in different countries (3). With Fig. 1, a very partial listing of some of these mechanisms is provided, a listing that is neither exhaustive nor explicit on the many submechanisms that lay behind each of the larger mechanisms. This figure, simplified though it is, still makes it clear that the task is a daunting one.

This latest finding on rising earnings inequality builds upon a preexisting country-specific literature showing that between-workplace or between-firm inequality accounts for a rising share of inequality in the United States, West Germany, and Sweden. The key contribution of Tomaskovic-Devey et al. is to show that this result is a very common one that holds in 12 of 14 well-off countries.

In each of the panels of this figure, we distinguish between a baseline condition and “time 2,” with the conceit that time 2 captures the effect of an ongoing process or intervention, such as a reduction in institutional protection. The markers refer to individual workers who differ in their earnings and are dichotomized into high-skill (red) and low-skill (blue) classes. The baseline condition allows for only two firms, but new firms are allowed at time 2 when the interventions at hand entail them. Within each firm, the vertical axis may be assumed to refer to skill as well as to earnings, an assumption that implies that within-firm skill and earnings rankings are the same. We also allow for the possibility, however, that some firms provide a premium to all employees, a premium that we will interpret as evidence of rent rather than, say, compensating differentials (in which a firm compensates for unpleasant conditions with extra pay). Following convention, “rent” refers to a payment in excess of what is needed to keep a factor of production, such as labor, engaged in the market.*

Based on this toy setup, we can straightforwardly distinguish among six different mechanisms, each of which yields an increase in between-firm earnings. The first diagram (Fig. 1 *A*, *Left*) represents a baseline condition in which Firms A and B pay equivalent workers the same amount. Although Firm A has higher average pay, this is simply because it hires higher-skill workers. Between the baseline and time 2, we introduce a shift in the environment that 1) allows Firm A to secure supernormal returns (perhaps because of market consolidation) and 2) results in rent sharing with its workers. This in turn generates an increase in both between-firm inequality and total inequality. If we sought a cause-targeted reform under this scenario, it would presumably involve reducing consolidation or otherwise reducing the capacity of firms to extract rent.

In the next diagram (Fig. 1 *A*, *Center*), our new baseline condition is the same as the time 2 condition in the preceding panel, meaning that we are still presuming that Firm A collects rent and then shares it with its employees. The time 2 condition for this diagram presumes a skill-biased shift in capacities for geographic mobility (i.e., low-skill workers come to be “locked in place”) that brings about a re-sorting in which high-skill workers increasingly work for high-wage firms. As shown here, this re-sorting increases between-firm inequality, as low-skill workers are no longer able to move to high-premium firms (i.e., Firm A), while high-skill workers can more readily take a position in these rent-collecting firms. Per the locked-in-place story line, we might assume that high-skill workers have the wealth to move to jobs in urban centers, where high-premium firms are increasingly found. Under this re-sorting, the total amount of rent stays the same, but it is now distributed in a way that is inequality enhancing (because high-skill workers are increasingly likely to be beneficiaries).

The next diagram (Fig. 1 *A*, *Right*) starts with a baseline in which both Firm A and Firm B benefit equally from rent (and again we assume it is shared with workers). However, average earnings are lower in Firm B, because it employs more low-skill workers. For this panel, our purpose is to illustrate the classic outsourcing narrative, instigated by a decline in worker protection that allows firms to lay off their low-skill workers and contract with other firms (i.e., “Out A” and “Out B”) for the same services. This turn to outsourcing reduces the earnings of low-skill workers (as they lose their rent), reduces within-firm inequality (as low-skill workers exit Firms A and B), and increases between-firm inequality. The cause-targeted reform under this scenario is of course a ramp up in institutional protections [just as Tomaskovic-Devey et al. (1) discuss].

Unlike the foregoing scenarios, those in Fig. 1*B* do not posit firm premiums, and the associated cause-targeted reforms will not, therefore, involve eliminating firm rent (or changing the beneficiaries of firm rent). We begin, for example, with two scenarios in which firms become increasingly segregated by skill level (i.e., Fig. 1 *B*, *Left* and *Center*). The baseline for these scenarios shows Firm A with higher average earnings than Firm B because it hires more high-skill workers. We then posit that pressures for skill-based segregation intensify and that workers either sort into firms dominated by their skill level (i.e., Fig. 1 *B*, *Left*) or that firms subdivide along skill lines (i.e., Fig. 1 *B*, *Center*). There are many reasons why pressures for skill-based segregation of this sort might be intensifying. If a firm is located, for example, in an area with high housing costs, it may be strategic to hive off its low-skill workers into a separate establishment in a lower-cost area (i.e., Fig. 1 *B*, *Center*) or contract with another firm in that area for equivalent work (i.e., Fig. 1 *B*, *Left*). The important point for our purposes is that ramped-up worker protections may not be the right prescription for organizational problems of this particular sort (although they are likely desirable for all manner of other reasons).

The final diagram concludes with the standard-issue case of rising returns to skill. As shown here (Fig. 1 *B*, *Right*), high-skill workers in both Firms A and B secure a premium in time 2, thus increasing between-firm inequality (as Firm A has more high-skill workers than Firm B). Although here again we are seeing an increase in between-firm inequality, a ramp up of worker protection, as desirable as it may be, is not responding to the cause and may not fully address its implications.

The upshot is that much would be gained by building on the seminal contribution of Tomaskovic-Devey et al. (1) with a robust set of analyses of the mechanisms underlying the rise in between-firm (or between-workplace) inequality. If this next step were taken, we could proceed with cause-targeted reform with more assurance. The good news is that there are many powerful approaches that may be used to sort out causes (3).

## The Consequences of Rising Between-Firm Inequality

Although we have focused to this point on the importance of sorting out causes, it is arguably just as important to understand the effects of rising between-firm inequality. Whatever the mechanisms behind the results of Tomaskovic-Devey et al. (1) may be, it is likely a pregnant fact that a growing share of total inequality occurs between workplaces. This result means that, just as neighborhoods are becoming increasingly segregated in some countries, so too workplaces are become increasingly segregated.

Why might this matter? Most obviously, it works to constrict social networks, potentially compromising the amount and quality of information that is received at the workplace. Although such a constriction may protect workers from invidious comparisons with those who earn more, it can also support various types of myopia and poor decision making. The rise of the earnings-segregated workplace may explain, at least in part, why workers often underestimate the extent of inequality (7). It may further affect the flow of opportunities. If low-skill workers are increasingly working in skill-homogeneous firms, it reduces their exposure to other occupations, reduces skill transfers from high-skill workers, and reduces access to friendships with them.

These hypotheses, which are but a sampling of possible effects, are of course wholly speculative and only intended to highlight that rising workplace segregation may affect many outcomes besides earnings inequality.

## Footnotes

*For the purposes of this discussion, we refer mainly to between-firm inequality (rather than between-workplace inequality), because this allows us to draw on a richer set of mechanisms.
